# Processing of Damaged DNA Ends for Double-Strand Break Repair in Mammalian Cells

**DOI:** 10.5402/2012/345805

**Published:** 2012-12-04

**Authors:** Lawrence F. Povirk

**Affiliations:** Department of Pharmacology and Toxicology, Virginia Commonwealth University, P.O. Box 980035, Richmond, VA 23298, USA

## Abstract

Most DNA double-strand breaks (DSBs) formed in a natural environment have chemical modifications at or near the ends that preclude direct religation and require removal or other processing so that rejoining can proceed. Free radical-mediated DSBs typically bear unligatable 3′-phosphate or 3′-phosphoglycolate termini and often have oxidized bases and/or abasic sites near the break. Topoisomerase-mediated DSBs are blocked by covalently bound peptide fragments of the topoisomerase. Enzymes capable of resolving damaged ends include polynucleotide kinase/phosphatase, which restores missing 5′-phosphates and removes 3′-phosphates; tyrosyl-DNA phosphodiesterases I and II (TDP1 and TDP2), which remove peptide fragments of topoisomerases I and II, respectively; and the Artemis and Metnase endonucleases, which can trim damaged overhangs of diverse structure. TDP1 as well as APE1 can remove 3′-phosphoglycolates and other 3′ blocks, while CtIP appears to provide an alternative pathway for topoisomerase II fragment removal. Ku, a core DSB joining protein, can cleave abasic sites near DNA ends. The downstream processes of patching and ligation are tolerant of residual damage and can sometimes proceed without complete damage removal. Despite these redundant pathways for resolution, damaged ends appear to be a significant barrier to rejoining, and their resolution may be a rate-limiting step in repair of some DSBs.

## 1. Introduction

DNA double-strand breaks are extremely toxic DNA lesions that arise from a variety of sources, including ionizing radiation [1], radiomimetic drugs [2, 3], oxidative stress [4, 5], abortive or inhibited topoisomerase reactions [6], and immunological processes such as V(D)J and class-switch recombination [7]. Thus, DSB repair is a critical process to which mammalian cells have devoted enormous resources, creating a complex network of repair systems that are intricately linked with cell cycle control and survival/death pathways [7]. Remarkably, molecular mechanisms of DSB repair in mammalian cells almost completely eluded researchers for decades, until the implication of Ku autoantigen in 1994 [8] unleashed a cascade of investigations by which the major players and primary mechanistic details of DSB rejoining were rather rapidly defined. 

Much of the seminal work elucidating these repair systems has taken advantage of defined DSB substrates, either constructed *in vitro* or formed in cells by site-specific nucleases [9]. These defined DSBs typically have canonical 5′-phosphate and 3′-hydroxyl termini suitable for further processing by exonucleases, polymerases, and ligases. Thus, this experimental approach, while extremely powerful, bypasses an important step in repair, namely, the cleanup of the chemically modified termini and/or damaged bases that accompany most DSBs formed in a natural or clinical environment. Several enzymes have been described that are capable of resolving various end modifications, and their specificity and cofactor requirements have been determined in some detail. A limited number of studies have been directed toward distinguishing which of these enzymes are actually used for repair in cells, and much work remains to be done in this area. Other cellular studies, however, suggest that resolution of damaged ends, especially those with complex or multiple modifications, can be a critical and in some cases rate-limiting step in repair [10]. This paper will attempt to summarize current knowledge of the molecular mechanisms for resolution of damaged DSB ends in mammalian cells and the biological consequences of that processing. Studies based on other organisms such as yeast are included only insofar as they provide insight into questions for which no comparable mammalian data are available. 

## 2. Structural Damage at DSB Ends 

### 2.1. Radiation-Induced and Other Free Radical-Mediated DSBs

**Figure 1:**
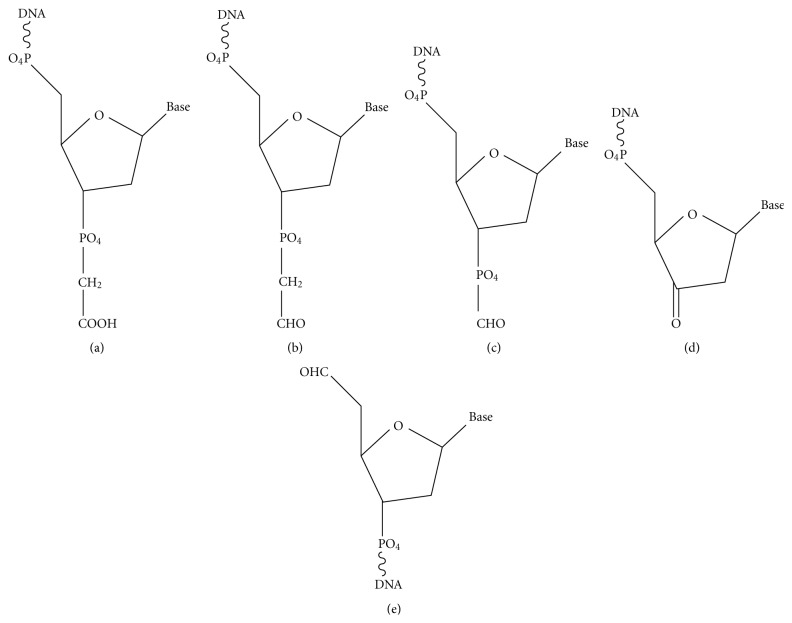
Some of the major damaged termini of free radical-mediated DNA strand breaks. (a) 3′-phosphoglycolate (PG). (b) 3′-phosphoglycoaldehyde. (c) 3′-formyl phosphate. (d) 3′-keto-2′-deoxynucleotide. (e) 5′-aldehyde.

DSBs induced by ionizing radiation stem from attack by free radicals, primarily the hydroxyl radical, on deoxyribose, with formation of carbon-centered free radicals on any of the five deoxyribose carbons potentially leading to strand cleavage. Significant free radical-induced blocked termini include nucleoside 5′-aldehyde on the 5′-side, and phosphoglycolate (PG), phosphoglycoaldehyde, formyl phosphate, and 3′-keto-2′-deoxynucleotide on the 3′-side [1, 11, 12] (Figure 1). Most of these moieties, as well as most of the free radical-generated oxidized abasic sites, are unstable and break down spontaneously to leave breaks with 3′- and 5′-phosphates. A notable exception is the 3′-PG, whose stability against further degradation even under harsh conditions [13] has rendered it the probe of choice for studies of 3′-end-processing. However, the 2′-oxidized abasic site is considerably more stable than other abasic lesions [14], and both this lesion and the 5′-aldehyde [15] can readily be isolated intact from treated DNAs. The relative frequencies of the various modified termini are presumably similar for single versus double strand breaks, but there are few quantitative data available on this point. An early study utilizing end-labeled DNA suggested that approximately half of all radiation-induced breaks bore 3′-PG termini [16], with 3′-phosphates comprising most of the remainder. However, a more recent measurement by mass spectrometry suggested a lower 3′-PG incidence of approximately 10% of total sugar oxidation products [17]. 

Considerable attention has been devoted recently to clustered damage induced by “spurs” of closely spaced ionizations that surround tracks of the secondary electrons dislodged by *γ*-rays [25, 26]. Such lesions presumably contain random mixtures of strand breaks, abasic sites, and any of the myriad forms of oxidative base damage, including 8-oxoguanine (8-oxoG) and thymine glycol [1, 27]. Monte Carlo calculations of radiation tracks predict that a substantial portion of damage sites will harbor multiple lesions, including DSBs with accompanying nearby base damage [28, 29]. The primary empirical evidence for clustered lesions is the generation of additional DSBs in irradiated DNA or cells by posttreatment with glycosylases and abasic endonucleases that together cleave sites of base damage [4, 30]. While these data clearly confirm that complex lesions do occur, biochemical studies with purified glycosylases and defined synthetic substrates indicate that nearby breaks and gaps can block recognition and removal of damaged bases [31–33], suggesting that glycosylases require a relatively intact duplex DNA structure on which to act. Thus, base damages near DNA ends, even if not sequestered by DSB repair proteins, are likely to be poor substrates for repair glycosylases and would instead have to be removed by DSB-specific mechanisms such as end trimming. 

Similar to radiation, the radiomimetic natural products bleomycin, neocarzinostatin (Zinostatin), and calicheamicin induce DSBs by free radical mechanisms, but the damage is largely restricted to the deoxyribose sugar moiety and to a few specific carbons therein [2, 3]. The Zinostatin- and calicheamicin-induced DSBs are similar in chemical structure but are formed on a 2-base (Zinostatin) and 3-base (calicheamicin) 3′-stagger between breaks in opposite strands. For either compound, one DSB end has a 5′-aldehyde and mixture of 3′-phosphate and 3′-PG termini on a 1-base (Zinostatin) or 2-base (calicheamicin) 3′-overhang. The opposite DSB end has both 5′- and 3′-phosphate termini, on a 2-base (Zinostatin) or 3-base (calicheamicin) 3′-overhang. Bleomycin induces DSBs with predominantly blunt ends or 1-base 5′-overhangs, with predominantly 3′-PG termini [34], owing to its specifically targeting oxidation of the C-4′ position, from which 3′-PGs derive [35]. 

For diffusible oxidants such as H_2_O_2_, strand breaks result principally from Fenton reactions involving Fe^++^ ions bound to DNA [36]. While the chemistry of DNA cleavage is similar to that seen with radiation, attributable mostly to oxidative fragmentation of deoxyribose by attack of hydroxyl radicals [37], there are marked sequence preferences for cleavage, owing to preferential Fe^++^ binding at certain sites on DNA [38]. Presence of at least 25% 3′-PG termini was reported for strand breaks in DNA from H_2_O_2_-treated cells [39], and this fraction would likely be similar for SSBs and DSBs. However, the initial ratio of SSBs to DSBs is much higher for H_2_O_2_ (~500 : 1) [40] than for radiation (~25 : 1) [41]. Thus, most DSBs in H_2_O_2_-treated cells result from collision of SSBs with replication forks. DSBs in nonreplicating G1 cells are much less frequent but they do occur [5], possibly as a result of local repetitive redox cycling of DNA-bound Fe^++^ [36] or by collision of SSBs with transcription bubbles and subsequent oxidative or endonucleolytic cleavage of the exposed single-stranded DNA. 

### 2.2. Topoisomerase-Mediated DSBs

**Figure 2:**
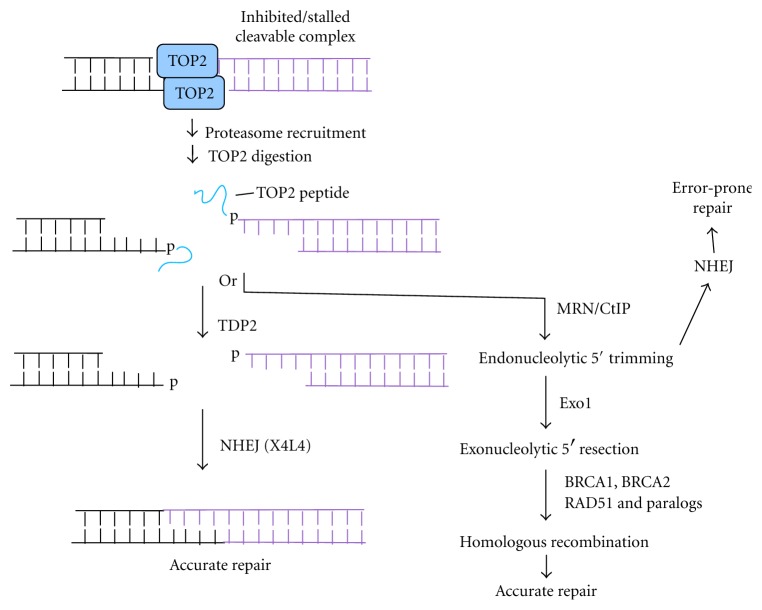
Formation and resolution of TOP2-mediated DSB ends. Proteasome recruitment to a persistent TOP2 cleavable complex results in digestion of covalently linked TOP2 to a short peptide, which can be removed by TDP2 to leave cohesive ligatable ends that can be accurately rejoined by NHEJ. Alternatively, CtIP-dependent endonucleolytic cleavage, perhaps catalyzed by MRE11 in its complex with RAD50 and NBS1 (MRN), can lead to either error-prone NHEJ of the resulting noncohesive ends or to 5′→3′ resection by exonuclease 1, culminating in HRR.

**Figure 3:**
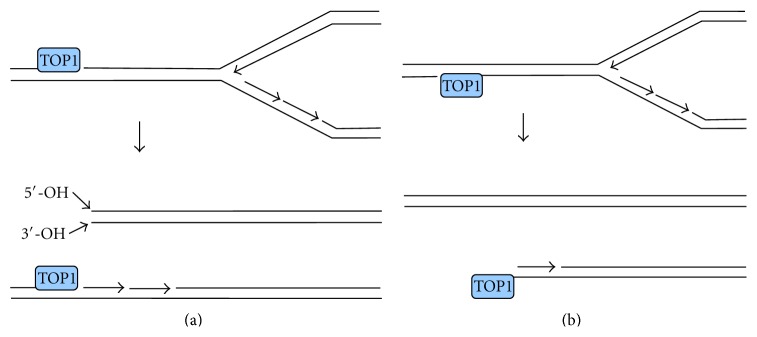
Formation of one-ended DSBs by collision of replication forks with persistent TOP1 cleavable complexes. (a) Collision with a lesion in the leading template strand results in a DSB with both 5′- and 3′-hydroxyl termini, plus a residual 3′-TOP1-terminated SSB. (b) Collision with a lesion in the lagging template strand results in a one-ended DSB with a TOP1-linked 3′-terminus and an Okazaki fragment at the 5′-terminus.

DNA topoisomerases I and II (TOP1 and TOP2) relax DNA by inducing transient SSBs (TOP1) and DSBs (TOP2) wherein the topoisomerase is covalently linked through a tyrosine to DNA 3′-phosphate (TOP1) or 5′-phosphate (TOP2) termini [42, 43]. Normally the breaks are rapidly rejoined by the topoisomerase with concomitant dissolution of the tyrosine-DNA covalent bond, but reversal can be prevented by inhibition or inactivation of the enzyme, or by oxidative damage to the DNA. In the case of TOP2 (Figure 2), the irreversible DSB will then have 3′-hydroxyl termini, and 4-base 5′-overhangs terminated in a tyrosyl-linked topoisomerase [42]. TOP1-mediated DSBs arise primarily by collision of replication forks with SSBs, resulting in a so-called “one-ended” DSBs [43]. These DSBs would likely have normal 3′-hydroxyl and 5′-hydroxyl termini and blunt ends if formed from a SSB in the leading template strand, or 5′-phosphate and 3′-TOP2-linked termini, if formed from a SSB in the lagging template strand, although neither of these structures has been verified empirically (Figure 3). Moreover, DSBs can also be formed by a replication-independent but transcription dependent process, probably involving collision of a transcription complex with a SSB [6, 44]. These DSBs are correlated with formation of R-loops (locally denatured DNA segments with one strand partially annealed to nascent mRNA) in the wake of arrested transcription complexes. However, the mechanism by which R-loops promote DSBs is currently unknown, and thus the structure of these DSBs is difficult to predict. It is possible that the breaks in both DNA strands are nucleolytic and occur some distance from the initiating TOP1-mediated SSB. 

Particularly when considering the repair of damage induced by topoisomerase inhibitors, it is important to distinguish between irreversible breaks and cleavable complexes. The latter are merely intermediates in DNA relaxation that are stabilized by the inhibitor [45, 46]. While the DNA strands in cleavable complexes are indeed broken and are detected as breaks upon detergent lysis of the cells, they are rapidly religated by the topoisomerase if instead the inhibitor is simply removed. When replication forks or transcription complexes collide with cleavable complexes that are persistent due to inhibitors, oxidative DNA damage, or topoisomerase inactivation, proteasomes are recruited to degrade the topoisomerase [47–50], resulting in an irreversible break with a topoisomerase fragment linked to one DNA terminus (Figure 2), which forms the actual substrate for the end processing enzymes discussed in the following sections. 

## 3. End Processing Requirements in Nonhomologous End Joining (NHEJ) and Homologous Recombination Repair (HRR)

**Figure 4:**
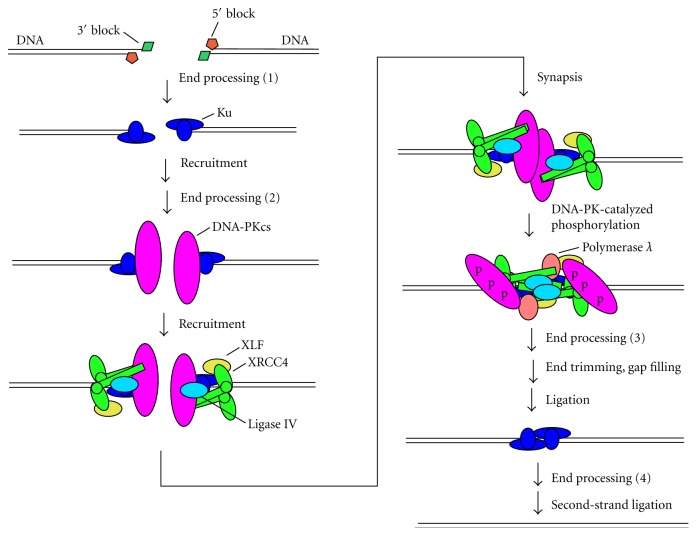
Resolution of damaged termini in NHEJ. Ku binds to DNA ends and recruits DNA-PKcs, the XRCC4/DNA ligase IV complex, and XLF. Synapsis of two DSB ends triggers DNA-PKcs autophosphorylation in *trans*, inducing a conformational change that allows gap filling by polymerase *λ* and finally ligation by DNA ligase IV. Removal of 5′- and 3′-terminal blocks by end processing enzymes can potentially occur at several steps in the pathway: before recruitment of any NHEJ factors (1); after recruitment of Ku (2) (e.g., by Ku itself [18]); or after DNA-PKcs autophosphorylation (3), which increases accessibility of the ends. In addition, processing of terminal blocks in the second strand can occur after ligation of the first strand (4), possibly by SSB repair pathways after dissociation of NHEJ proteins.

**Figure 5:**
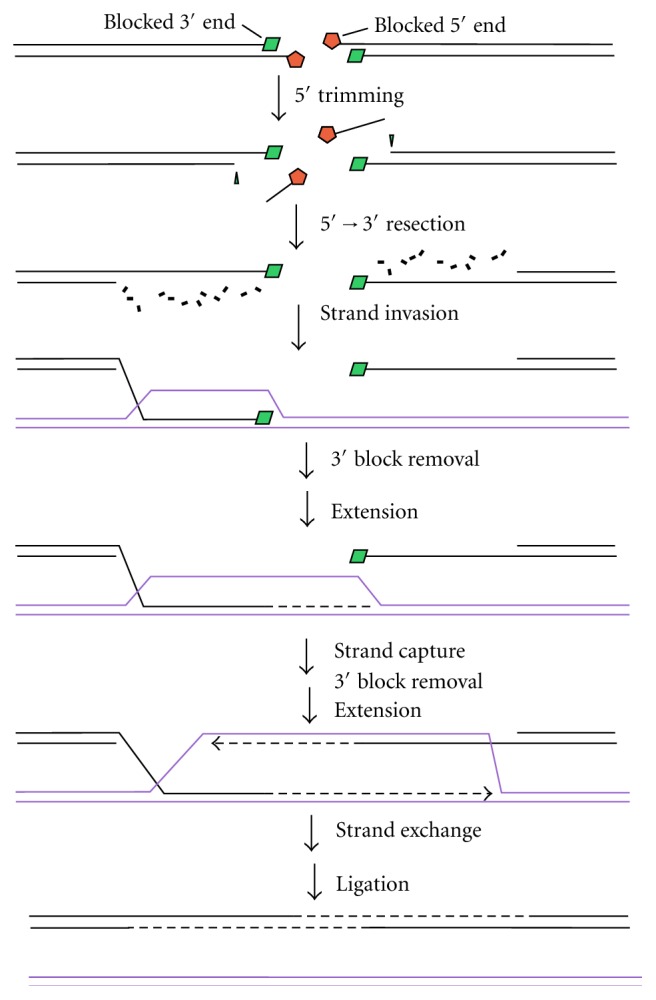
Resolution of damaged termini for HRR. In HRR, following its initiation by MRN/CtIP, 5′→3′ exonucleolytic resection generates 3′-overhangs, one of which invades a sister duplex of the same sequence. Extension of this 3′-end (dashed line) enlarges the D-loop until it can be captured by the other 3′-overhang. Extension of this overhang followed by resolution of crossover structures restores an intact DNA duplex. Blocked 5′-termini must be removed before 5′→3′ exonucleolytic resection, probably by CtIP-dependent endonuclease activity. Removal of blocked 3′-termini could occur either before or after strand exchange but must occur before the respective extension steps.

Consideration of the resolution of damaged DSB ends must take into account the two distinctly different mechanisms by which DSBs are repaired in mammalian cells. In NHEJ, 5′- and 3′-termini are in general minimally processed to yield ends that can be annealed or juxtaposed, then patched and ligated (Figure 4). Thus, at least one strand must have a 3′-hydroxyl terminus suitable for polymerase-mediated extension and ligation on one end and a 5′-phosphate on the other end. Given the relatively high tolerance of the XRCC4/DNA ligase IV (X4L4) complex for imperfectly matched ends (see Section 5), the other strand could in principle bear unligatable termini (although probably not large adducts), which could be resolved after ligation of the first strand. However, at least in some cases, enzymes capable of removing damaged termini on either recessed or protruding 5′- or 3′-single strands would be required. Such terminal processing could occur at several points in the NHEJ pathway (Figure 5), for example, even before Ku binding, or after synapsis and DNA-PK autophosphorylation. Moreover, processing of blocked termini on the second strand could occur after ligation of the first strand and dissociation of the NHEJ repair complex. 

HRR, on the other hand, involves extensive exonucleolytic 5′-resection, followed by invasion of a homologous sister duplex by the exposed 3′-overhang [51]. In this case, removal of any 5′-blocks, at both ends of the break, is essential at a very early step in repair (Figure 5). The strand invasion step would likely proceed despite small chemical modifications of the 3′-overhang, although any blocked 3′-termini would have to be removed prior to the extension step. 

## 4. DNA End-Processing Enzymes Acting at Blocked DSB Ends

### 4.1. Tyrosyl-DNA Phosphodiesterases

Tdp1 was isolated biochemically as a yeast enzyme that removed a protein fragment of TOP1 from the 3′-end of DNA [52]. The gene was identified by random mutagenesis [53], and the corresponding human gene was soon cloned by homology [54]. *In vitro*, human TDP1 removes tyrosyl-linked peptides as well as simple tyrosyl moieties from 3′-termini of either SSBs or DSBs, leaving a 3′-phosphate that can then be removed by PNKP (see Section 4.3). TDP1 is also capable of removing other 3′-blocks such as 3′-PGs [55] and cleaved abasic sites [56], although less efficiently than 3′-tyrosyl linkages; for example, human TDP1 removes 3′-phosphotyrosyl moieties about 100 times more efficiently than 3′-PGs [55]. The active site of the enzyme contains a putative binding channel for single-stranded DNA [57], suggesting that double-stranded substrates become partially denatured prior to phosphotyrosyl cleavage. As predicted from such a model, TDP1 acts more efficiently on single-strand than on double-strand substrates [58] and more efficiently on single-strand 3′-overhangs than on 3′-recessed ends of DSBs [59]. 

The extremely rare human genetic disease spinocerebellar ataxia with axonal neuropathy (SCAN1) is associated with an H493R mutation in TDP1 [60] that reduces the rate of hydrolysis of tyrosyl-DNA bonds by about 25-fold, and also dramatically increases the lifetime of a transient intermediate wherein TDP1 is covalently linked to the DNA 3′-terminus [61]. Despite the leakiness and complexity of this mutation, these cells provide a model of TDP1 deficiency for assessing its role in repair of various DNA lesions. TDP1-deficient mice, murine embryonic fibroblasts (MEFs) [62–64], and DT-40 chicken erythrocytes [65] have also been generated. There is little doubt that TDP1 is instrumental in removing 3′-linked TOP1 fragments from SSBs, as SSBs are more persistent in SCAN1 cells than in normal cells after treatment with TOP1 inhibitors [66]. Moreover, TDP1 binds to DNA ligase III, which carries out the ligation step in SSB repair [66]. Conversely, there is no evidence of TDP1 binding to any NHEJ or HRR proteins nor any evidence that TDP1 is recruited to DSB ends by other DSB repair factors. In fact, *in vitro* NHEJ proteins inhibit TDP1 activity toward DNA ends, probably by restricting access to the termini [59]. As judged by *γ*H2AX focus-formation assays, there is no detectable effect of TDP1 deficiency on repair of radiation-induced DNA DSBs in either SCAN1 cells [67] or MEFs [63]. The slight sensitivity of SCAN1 cells to radiation is apparently attributable to a defect in repair of SSBs [67]. Tdp1^−/−^ MEFs and DT-40 cells [65] as well as SCAN1 cells [68] are sensitive to the TOP1 inhibitor camptothecin, but this sensitivity is probably likewise mostly due to a defect in SSB repair, and it is unclear whether TDP1 is involved in TOP1 removal from the DSBs.

Nevertheless, Tdp1^−/−^ mice [62] and Tdp1^−/−^ DT-40 cells [65] are both sensitive to bleomycin, while TDP1-knockdown HeLa cells are slightly sensitive to calicheamicin [59]. Moreover, following treatment with calicheamicin, SCAN1 cells show more chromosome aberrations, particularly dicentric chromosomes, than normal cells [59]. In extracts of SCAN1 cells [69] and Tdp1^−/−^ MEFs [64], PG termini on 3′-overhangs of DSBs are highly persistent, with no detectable processing for several hours, while in extracts from normal cells, substantial processing can be seen within minutes. Thus, despite the fact that TDP1's activity toward PG termini is about 100 times weaker than toward its canonical 3′-phosphotyrosyl substrate [55], all these results suggest a major role for TDP1 in resolution of 3′-PG termini of DSBs, on both blunt ends and 3′-overhangs. With calicheamicin in particular, it is unlikely that the observed sensitivities result from effects on SSB repair, as nearly all lesions induced by calicheamicin are bistranded [70]. 

A role for TDP1 in resolution of TOP2-linked DSBs has been controversial. Neither yeast Tdp1 nor human TDP1 [71] has any detectable activity toward a simple 5′-phosphotyrosyl oligonucleotide. However, a more realistic substrate consisting of an oligonucleotide with phosphotyrosyl-linked peptide, derived from a TOP2 cleavable complex, is cleaved by yeast Tdp1 [72] and less efficiently by human TDP1 (J. L. Nitiss, University of Illinois college of Pharmacy, personal communication). SCAN1 cells [68] and Tdp1^−/−^ mice [62] show no sensitivity to TOP2 inhibitors. However, Tdp1^−/−^ DT-40 cells are sensitive to TOP2 inhibitors [65], while overexpression of TDP1 in 293 cells reduces the level of DNA damage seen after TOP2 inhibitor treatment [73]. Overall, the results suggest that under some conditions TDP1 can resolve TOP2-linked DSBs. Apparent species-specific differences may reflect the differences in the efficiency of alternative repair pathways for these lesions. 

TDP2, which has no homology to TDP1, has robust phosphodiesterase activity toward 5′-tyrosyl DNA ends and much weaker activity toward 3′-tyrosyl ends [71]. Tdp2^−/−^ MEFs and DT-40 cells show significant sensitivity to TOP2 inhibitors [74, 75]. Thus, TDP2 apparently plays a major role in resolution of TOP2-linked DSBs (Figure 2), although increased persistence of TOP2-linked DNA in TDP2-deficient cells has yet to be directly demonstrated.

### 4.2. CtIP

CtIP is a critical factor in channeling DSBs to either the NHEJ or the HRR pathway. In late S and G2 phase, CtIP phosphorylation at S327 by CDK2 promotes BRCA1 recruitment and the initiation of 5′-resection, which in turn precludes NHEJ and commits a DSB to repair by HRR [76–78]. However, CtIP also has modulatory effects on NHEJ even in G1, where it promotes certain microhomology-mediated end joining (MMEJ) events [77]. Sae2, the *S. cerevisiae* homologue of CtIP, harbors endonuclease activity that acts on overhangs near hairpins [79, 80], and it has been proposed that this activity initiates 5′-resection for HRR by endonucleolytically releasing an oligonucleotide (10–40 bp) from the 5′-end [81]. Such Sae2-dependent cleavage can most clearly be seen in the processing of SPO11-linked breaks generated during meiosis [82], and similar cleavage can be seen in mouse testes, presumably by either an endonucleolytic activity of CtIP or a CtIP-dependent activity of MRE11. An initial report of MRE11-dependent CtIP nuclease activity *in vitro* [76] has yet to be further elucidated. 

Human fibroblasts deficient in either CtIP or MRE11 show a profound deficiency in repair of DSBs induced by the TOP2 inhibitor etoposide in G1 phase, as judged by *γ*H2AX focus formation [83]. Similar results were seen when CtIP was knocked down by siRNA in either fibroblasts or HeLa cells. Inasmuch as the repair was shown to be ligase IV-dependent, these results suggest that both CtIP and the MRE11/RAD50/NBS1 (MRN) complex are required for NHEJ of TOP2-linked DSBs, most likely reflecting a role in endonucleolytic removal of the TOP2 peptide from the 5′-ends of the DSBs (Figure 2). Remarkably, in these studies there was virtually no repair in CtIP-knockdown fibroblasts even as long as 6 hr after treatment. It seems surprising that TDP2 apparently could not substitute, even partially, for CtIP in resolving these blocked DSB ends. 

CtIP-deficient mouse cells [84] and DT-40 cells [85] are both inviable. However, DT-40 cells harboring an unphosphorylatable S332A CtIP allele are viable and proficient in homologous recombination but are sensitive to both TOP1 and TOP2 inhibitors [85]. Since the S332A mutation abrogates CtIP binding to Brca1, these results suggest that Brca1 and CtIP are both required for endonucleolytic release of both TOP1 and TOP2 fragments from 3′- and 5′-termini, respectively, of topoisomerase-mediated DNA breaks, although again increased persistence of topoisomerase-linked DNA was not explicitly demonstrated. Curiously, while CtIP(S322A)/Tdp1^−/−^ DT-40 cells were much more sensitive than either single mutant to camptothecin, the two mutations were epistatic in conferring sensitivity to etoposide [65]. These results suggest that CtIP and Tdp1 are essential factors in a single pathway for resolution of TOP2 DSBs, a conclusion that is somewhat difficult to rationalize in terms of their known biochemical activities. Moreover, the inference that this DSB repair requires CtIP phosphorylation at S332 (equivalent to human S327) and CtIP binding to Brca1 is difficult to reconcile with the human cell studies, wherein cells expressing an S327A mutant of CtIP had wild-type proficiency in G1 repair of etoposide-induced DSBs [83]. 

### 4.3. Polynucleotide Kinase/Phosphatase (PNKP)

PNKP removes phosphates from 3′-DNA ends and phosphorylates 5′-ends, using ATP as a cofactor [86, 87], but has no other known activities toward other modified termini, including 3′-PGs [55]. PNKP shows no strict dependence on DNA secondary structure, acting on simple oligomers, as well as nicks, gaps, and DSB ends. PNKP binding to XRCC1 [88] and XRCC4 [89] suggests recruitment to repair complexes for SSB and DSB repair, respectively, although recruitment to SSBs does not require and may precede XRCC1 recruitment [90]. shRNA-mediated knockdown of PNKP in A549 lung tumor cells confers moderate radiosensitivity [91]. This effect most likely reflects increased persistence of 3′-phosphates. 

### 4.4. Apurinic/Apyrimidinic Endonucleases

Other than apurinic/apyrimidinic (AP) lyases, human cells contain two known AP endonucleases, APE1 and APE2, that cleave the phosphodiester bond between the 5′-phosphate of the AP site and the preceding nucleotide, leaving a 3′-hydroxyl end [92]. Both enzymes are homologous to *E. coli* exonuclease III and have similar activities of AP endonuclease, 3′→5′ exonuclease, and a phosphodiesterase activity that removes 3′-PG and 3′-phosphate termini [92–94]. The canonical and by far the most efficient activity of APE1 is the cleavage of AP sites in duplex DNA. APE1 also removes 3′-PGs and other 3′-blocks from DNA ends, although its activity (measured as *k*_cat_/*K*_*m*_) toward 3′-PGs at internal nicks, recessed 3′-ends, and blunt ends is 100, 500, and 1800 times lower, respectively, than its abasic endonuclease activity [95]. Further, APE1 has no activity toward PGs on 3′-overhangs, even 1-base overhangs. Thus, the substrate preferences of APE1 are complementary to those of TDP1, which acts more efficiently on overhangs [59]. 

Studies of APE1 function have been complicated by the fact that it is essential for survival. Ape1-deficient mice and MEFs are inviable [96], and conditional Ape1^−/−^ cells die within days of Ape1 deletion [97]. However, APE1 knockdown renders TK6 lymphoblastoid cells and HCT116 colorectal carcinoma cells more resistant to radiation, but more sensitive to the radiomimetic agent bleomycin [98]. To explain this paradoxical result, it was proposed that bleomycin sensitivity reflects a defect in removal of the predominant 3′-PG moieties at ends of (mostly blunt-ended) bleomycin-induced DSBs, while radioresistance may result from fewer complex lesions such as strand breaks with closely opposed strand breaks being converted to toxic DSBs when APE1 is knocked down. Thus, these results suggest that APE1's phosphodiesterase activity on 3′-PG DSB ends is biologically significant, despite its inefficiency. An early report of association between APE1 and the core NHEJ protein Ku [99] has been neither confirmed nor refuted. APE1 can also remove 3′-phosphotyrosyl moieties from a recessed 3′-end, but even less efficiently than 3′-PGs [100]. 

APE2 has much weaker AP endonuclease activity than APE1 [92]. However, it has robust 3′→5′ exonuclease activity toward mismatched 3′-terminal bases, as well as 3′-PG removal activity [93, 94, 101]. Both activities are significantly stimulated by proliferating cell nuclear antigen (PCNA), with which APE2 colocalizes at nuclear foci in cells following exposure to oxidative stress [101]. Thus, based on its known activities and its homology to APE1, APE2 is a candidate enzyme for resolving 3′-PGs and other 3′-blocks, although its detailed substrate requirements and the kinetic parameters for various substrates have not been rigorously defined. 

### 4.5. Artemis

**Figure 6:**
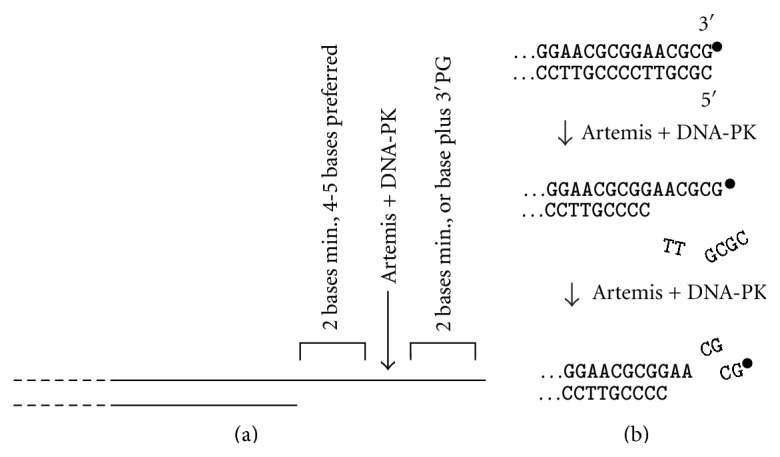
Trimming of DNA ends by Artemis nuclease. (a) Substrate specificity for trimming of 3′-overhangs. Long overhangs are trimmed 4-5 bases from the single-strand/double-strand junction. Shorter overhangs can be trimmed to as little as 2 bases, but 2 unpaired bases are required on each side of the cleavage site. A 3′-PG can substitute for the 3′-terminal base. (b) Trimming of blunt ends by Artemis. Several bases are first removed endonucleolytically from the 5′-terminal strand (which can be either phosphate- or hydroxyl terminated), generating a 3′-overhang that is then also trimmed, whether it is terminated in a 3′-PG (∙) or a 3′-hydroxyl. All endonucleolytic trimming by Artemis requires autophosphorylated DNA-PK.

Artemis was isolated as the factor mutated in a subset of human severe combined immune deficiency patients with accompanying radiosensitivity (RS-SCID) [102]. RS-SCID is also known as Athabascan SCID (SCID-A), owing to a single mutation detected with relatively high incidence in Athabascan Amerindians [103]. Artemis has intrinsic 5′→3′ exonuclease activity (recently suggested to be a contaminant [104]), but upon complexation with DNA-PK it acquires an endonuclease activity that opens DNA hairpins, which are formed as intermediates in V(D)J recombination. This same activity trims both 3′- and 5′-overhangs of DNA DSB ends, usually removing the 5′-overhang entirely while shortening 3′-overhangs to 4-5 bases [105] (Figure 6(a)). However, upon extended incubation, these short 3′-overhangs are further shortened to 2-3 bases [106]. Although only DNA-PKcs is strictly required for Artemis endonuclease activity, Ku enhances activity, especially for less favorable substrates, probably by improving DNA end binding [106]. 

Artemis is capable of trimming 3′-PG-terminated overhangs, thus providing a 3′-hydroxyl terminus for patching and ligation of DSBs, and on shorter overhangs (3–5 bases) the PG terminus stimulates trimming [106]. Artemis also coordinately trims both DNA strands at blunt ends, whether terminated in a 3′-PG or a 3′-hydroxyl [107] (Figure 6(b)). This process is much slower than the trimming of overhangs and proceeds via endonucleolytic removal of several bases from the 5′-terminal strand, followed by trimming of the resulting 3′-overhang. Whereas the 5′→3′ exonucleolytic activity of Artemis requires a 5′-phosphate terminus, this endonucleolytic trimming does not [107]. 

Thus, based on its biochemical properties, Artemis could resolve a 3′-PG, and probably any other small 3′-modification, in almost any context, including the 3′-overhanging PGs of Zinostatin- and calicheamicin-induced DSBs, as well as blunt-ended bleomycin-induced DSBs. Consistent with a significant role in DSB repair, RS-SCID patient-derived Artemis-deficient fibroblasts show increased sensitivity to Zinostatin, bleomycin, and ionizing radiation [106], as well as a defect in DSB repair following treatment with these agents [108]. Moreover, both hypersensitivity and DSB repair deficiency can be rescued by stable complementation with wild-type Artemis expressed from lentivirus, but not with endonuclease-deficient mutant of Artemis [108]. These results imply that radiosensitivity in Artemis-deficient cells reflects a defect in DNA processing, rather than the cell signaling functions of Artemis. 

In principle, Artemis could trim even the most extensively damaged DNA ends, for example, a radiation-induced DSB with multiple base damages near the end, and trimming might continue until an undamaged DNA segment is exposed. Base damage could promote single strandedness near the DNA end and thus promote Artemis-mediated cleavage, although these same structural modifications could have the opposite effect of interfering with substrate recognition. 

 However, implication of Artemis in trimming of modified DNA ends for DSB repair is complicated by the finding that the defect in repair is confined to a small fraction of the total DSBs, typically 10–20% in the case of radiation or Zinostatin [108, 109]. According to some studies, in Artemis-deficient cells, these breaks are never repaired, even after several days, while the other ~90% of DSBs are repaired as quickly as in normal cells [110]. The repair-resistant breaks appear to be primarily those in heterochromatin, and their repair also requires ATM, the MRE11/RAD50/NBS1 (MRN) complex, and 53BP1. A model has been proposed wherein both ATM and 53BP1 are required primarily to phosphorylate the heterochromatin protein KAP-1, resulting in decondensation of heterochromatin to allow access to DSB repair factors [109, 111]. Since Artemis and ATM are epistatic for repair of these slowly rejoined breaks [110], it may be inferred that Artemis, like ATM, is required only for repair of breaks in heterochromatin, although this has not been explicitly shown. Inasmuch as it is unlikely that the chemistry of the DSBs, especially the relatively well-defined breaks induced by Zinostatin and bleomycin [2, 3], is substantially different between heterochromatic and euchromatic breaks, it is difficult to explain why Artemis-mediated trimming of the DSB ends would be required only when the DSB is in heterochromatin. It is possible that the DSB repair mechanisms for persistent breaks such as those in heterochromatin are sufficiently different from those for rapidly repaired breaks and that enzymes which act on damaged ends are somehow excluded from acting on the more persistent DSBs. Alternatively, an intriguing study of DSBs induced at a putative “partially heterochromatic” DNA locus by the rare-cutting endonuclease I-SceI suggested that, rather than trimming DNA ends, Artemis excises an entire DSB-containing nucleosome, thus allowing rejoining of the more accessible linker regions on either side of that nucleosome and deletion of the DNA within the nucleosome [112]. 

With respect to a role for Artemis in repairing TOP2-linked DSBs, there are conflicting data on the effect of Artemis deficiency on sensitivity to TOP2 inhibitors. Homologous knockout of Artemis conferred significant (~2-fold) etoposide sensitivity to Nalm-6 pre-B cells [113], while knockout in HCT116 colorectal carcinoma cells had no effect on sensitivity [112]. RS-SCID patient-derived, Artemis-deficient fibroblasts showed no defect in rejoining of etoposide-induced DSBs [110], although in separate study, similar patient-derived cell lines showed slight sensitivity to etoposide [114]. A 3′-phosphotyrosyl-terminated 3′-overhang was cleaved, albeit rather slowly, by Artemis in presence of DNA-PK [106], but DNA ends bearing 5′- or 3′-phosphotyrosyl-linked protein fragments (i.e., structural models of topoisomerase-mediated DSBs) have apparently not been tested as Artemis substrates.

### 4.6. Metnase

Metnase, also called SETMAR, has both protein methyltransferase and endonuclease activities and was discovered in a search for human proteins homologous to bacterial transposases [115]. Although the nuclease activity of Metnase does not open DNA hairpins and does not require any protein cofactors, its specificities are otherwise remarkably similar to those of Artemis. It is inactive toward intact double-stranded DNA, but it trims both 5′- and 3′-overhangs of DSBs, as well as flaps and Y-structures mimicking frayed DSB ends [116]. It appears to require a free 5′- or 3′-DNA terminus for entry, as it does not cleave single-stranded loops flanked at both ends by double-stranded regions. Its methyltransferase activity promotes NHEJ, at least in part by methylating histone H3 at Lys36 [117]. However, its activity in stimulating and improving the fidelity of end joining of transfected plasmid substrates is dependent on its nuclease activity [118]. Thus, given its biochemical specificity, its binding to XRCC4, and its implication in NHEJ, Metnase is a candidate for trimming diverse types of damage to DNA ends, thereby creating substrates more amenable to patching and ligation. However, its activity toward damaged ends has yet to be explicitly investigated. 

### 4.7. Exonucleases

Mammalian cells contain a variety of 3′→5′ and 5′→3′ exonucleases that could in principle serve to remove terminal modifications, similar to exonuclease III in *E. coli *[119]. However, relatively few mammalian exonucleases have been tested for activity toward terminally modified substrates and those that have shown little such activity. The major 3′→5′ exonuclease activity in mammalian cell extracts is DNAse III [120], also called TREX1. Exonucleolytic digestion of DNA by DNAse III is completely blocked by a 3′-PG terminus, in either the presence or absence of Ku [121] or by a 3′-phosphotyrosyl terminus [100]. Its crystal structure revealed a tight nucleotide binding pocket that would be unlikely to either accommodate the extra bulk of a terminal PG or recognize the PG itself as a terminal nucleotide [122]. The Werner syndrome-associated protein WRN1, which binds to both Ku and XRCC4 [123] (suggesting some role in NHEJ), likewise has no activity toward either 3′-PG or 3′-phosphotyrosyl substrates [100]. Other mammalian 3′→5′ exonucleases, including RAD9 [124] and MRE11 [125] (which is strongly implicated in both NHEJ and HRR), do not appear to have been tested for activity toward modified termini.

Mammalian 5′→3′ exonucleases include Apollo (SNM1B, DCLRE1B), Artemis (SNM1C, DCLRE1B, discussed above), aprataxin, exonuclease 1 (EXO1), Fanconi-associated nuclease 1 (FAN1), flap endonuclease 1 (FEN1), and the aprataxin-and-PNKP-like factor (APLF, PALF) [126–128]. Aprataxin has been implicated in resolution of abortive 5′-adenylated ligase intermediates for both SSB repair and NHEJ [129]. In general, the 5′→3′ exonuclease function of the other enzymes either requires or strongly prefers a 5′-phosphate. Apollo, Artemis (discussed above), Aprataxin, exonuclease I, and PALF all have single-strand endonuclease activity as well that could in principle resolve either 3′- or 5′-terminal blocks. However, except for Artemis and aprataxin, there is only indirect evidence that resolution of damaged termini is a biologically relevant function of these enzymes in cells. PALF binds to XRCC4 and can trim 3′-overhangs to promote end joining in a defined *in vitro* NHEJ system based on purified recombinant proteins [130]. Fen-1 has been implicated in resolution of flap structures for NHEJ in yeast [131], but there is no comparable evidence in mammalian cells. Thus, overall there is little evidence for exonucleolytic removal of 5′-blocks. The mechanism by which the 5′-aldehyde termini of Zinostatin- and calicheamicin-induced DSBs are resolved is essentially unknown. One possibility is release of a 5′-terminal oligonucleotide by CtIP (see Section 4.2). Alternatively, as 5′-aldehydes are formed in only one strand of these DSBs, they could be removed by displacement synthesis following religation of the break in the complementary strand (see Section 5). 

### 4.8. Ku

Ku is a heterodimer that forms a basket-shaped structure whose “handle” threads onto DNA ends, making tight contact with the DNA grooves [132]. Once bound to a DNA end, Ku recruits other NHEJ factors [133] and stimulates the final religation step [134]. Ku also has a potent lyase activity that serves to cleave abasic sites near DNA ends [18]. This lyase activity also removes deoxyribose-5-phosphate moieties such as would be present at one 5′-terminus of a DSB formed by APE1-mediated cleavage of an AP site near a closely opposed strand break. Although lyase activity might be expected for any protein whose basic amine residues are in tight contact with DNA grooves, the lyase activity of Ku shows specificities which suggest that it may be specifically adapted to promote efficient and accurate NHEJ [135]. For example, it is more active toward AP sites on 5′-overhangs, where it would generate ligatable 5′-phosphates than on 3′-overhangs where a nonligatable cleaved sugar moiety would be left blocking the 3′-terminus. In addition, AP sites which are sufficiently distant from a terminus such that ligation can still occur despite their presence are relatively resistant to Ku's lyase activity. In other words, Ku appears to cleave AP sites preferentially in situations where doing so would facilitate end joining or increase its accuracy, whereas cleavage is suppressed when it would lead to unnecessary deletion of terminal nucleotides prior to religation. 

## 5. Damage Tolerance in NHEJ

**Figure 7:**
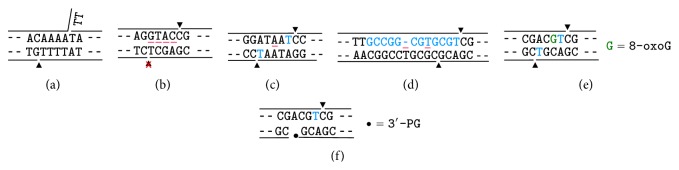
Tolerance for mismatched and damaged ends in patching and ligation for NHEJ. Black text shows sequences of ends prior to joining, while blue text shows fill-in of bases by DNA polymerase *λ*. Lines above and below the text represent the DNA backbone, triangles show sites of ligation by X4L4, and red bars between the bases show mismatches. All sequences read 5′→3′ in the top strand and 3′→5′ in the bottom strand. (a) Cohesive overhangs with an unpaired 3′-flap, ligated by X4L4 alone [19]. (b) Completely mismatched overhangs ligated in one strand only, by purified X4L4 plus Ku, DNA-PKcs, and XLF *in vitro* [20]. (c) Patching and ligation of annealed overhangs of two I-SceI-induced DSBs in hamster cells, despite an internal A-A mismatch, inferred from sequencing of repair joints [21]. (d) Ligation of a mismatched duplex resulting from error-prone fill-in in HeLa cell extracts supplemented with X4L4 and a highly error-prone mutant of polymerase *λ* [22]. The single dash in the top strand sequence indicates a single-base deletion. (e) Patching and ligation of partially complementary overhangs, one of which contains a 3′-terminal 8-oxoG, in X4L4-supplemented HeLa nuclear extracts [23]. (f) Patching and ligation of partially complementary overhangs in one strand, despite persistence of a 3′-PG-terminated break in the opposite strand, also in X4L4-supplemented HeLa nuclear extracts [24].

Like all DNA ligases, the NHEJ-associated DNA ligase IV has an absolute requirement for 5′-phosphate and 3′-hydroyxl termini. However, in its usual tight complex with XRCC4, ligase IV has considerable tolerance for ends that are not perfectly matched and overhangs that are not perfectly annealed. For example, short, partially complementary overhangs can be annealed and ligated despite the presence of single-stranded flaps, mismatched bases, and missing nucleotides in the presumed annealed segment (Figure 7). Moreover, the efficiency of ligation of imperfectly matched ends is increased markedly by the presence of XLF [20, 136, 137], a scaffold protein that forms filaments of alternating homodimers with XRCC4. *In vitro*, the combination of X4L4, Ku, DNA-PKCS, and XLF can join two ends with completely mismatched overhangs, albeit only in one strand (Figure 7(b)), and can also join a protruding 5′-overhang to a blunt end (not shown) [20]. The gap-filling NHEJ-associated DNA polymerase *λ* displays a similar tolerance for imperfectly annealed template/primers [138, 139]. While ligases and polymerases could in principle have higher stringency in the context of a full repair complex than when acting alone on a DNA substrate *in vitro*, end joining experiments in both extracts and intact cells confirm that ligation of mismatched overhangs can occur. For example, in Chinese hamster ovary (CHO) cells, end joining of two-ATAA 3′-overhangs of I-SceI-induced DSBs apparently proceeds predominantly by annealing the two terminal TAA trinucleotides to each other (despite the internal A•A mismatch), followed by single-base gap filling and ligation (Figure 7(c)) [21]. Further studies in human cell extracts supplemented with an extremely error-prone mutant form of polymerase *λ* likewise show that repair patches containing multiple mismatches can be ligated during NHEJ (Figure 7(d)) [22]. 

By analogy, it is likely that similar patching and ligation can occur even on ends that contain some degree of base damage and other modifications at or very near DNA termini. A few studies using defined substrates that incorporate such damage support this proposal. For example, similar to the -ATAA overhangs mentioned above, a substrate bearing at an -ACG 3′-overhang on one end and an -AC(8-oxoG) overhang at the other, can be annealed at the terminal dinucleotides, patched and ligated (Figure 7(e)). In this cell extract-based NHEJ model, the 8-oxoG-containing strand clearly undergoes polymerase *λ*-dependent single-base extension and ligation without prior removal of the damaged base [23]. However, presence of 8-oxoG on both overhangs appeared to block this process. Likewise, the nonplanar base thymine glycol suppressed patching and ligation of partially complementary -CTA overhangs by at least 90% [23]. 

This same *in vitro* system was able to join two ends, one with a PG-terminated -ACG 3′-overhang and one with an identical but hydroxyl-terminated overhang (Figure 7(f)). In this case, the hydroxyl-terminated strand was often patched and ligated, without any processing of the 3′-PG-terminated complementary strand [24]. With this substrate, the second, 3′-PG-terminated break could likely be repaired at a later time by the less error-prone base excision and SSB repair pathways. Similar postend-joining repair of residual damage could also occur for the DSB substrates containing abasic sites and damaged bases, presumably in a relatively error-free manner. Thus, in all these cases, tolerance for residual damage in end joining could actually increase fidelity, because a less tolerant system might require endonucleolytic trimming of these damaged ends, resulting in deletion of terminal nucleotides in the repaired products. 

In a different extract-based end joining assay, either 8-oxoG or a base mismatch at the penultimate base pair of a blunt-ended DSB was found to strongly inhibit end joining, while an abasic site the same position blocked joining entirely [140]. Although the specific substrates used were not identical, this experimental system appears to show somewhat less tolerance for nearby mismatches, abasic sites, and base damage than those mentioned above. Nevertheless, end joining products that retained damaged bases or base mismatches were detected in this system as well. 

## 6. Perspectives

Overall, mammalian cells are seen to harbor multiple redundant pathways and enzymes for resolution of damaged DSB ends, with diverse specificities and various degrees of sequence conservation. Much remains to be determined regarding which mechanisms predominate under particular circumstances and how the various pathways are prioritized in the cell. Experimentally, a major obstacle to addressing these issues is the lack of assays capable of tracking DSB end processing in intact cells. Technologies to induce terminally modified DSBs at a defined time and at specific sites would be very helpful in this regard. While chemical techniques such as mass spectrometry are still far too insensitive to be used to follow DSB end processing, continuing improvements and adaptations may yet make such direct chemical analysis tractable.

With respect to therapeutic implications, the most obvious application would be to identify inhibitors that would block end processing and thereby enhance the antitumor activity of DSB-inducing cancer chemotherapeutic agents. Given the redundancy of end processing pathways outlined above, resolution of damaged ends might seem an unlikely target for inhibition. Nevertheless, despite apparent redundancy, some of the single mutants, such as the Tdp2^−/−^ DT-40 cells and MEFs [74, 75], do show significant sensitivity to certain types of DSBs. Given the general tendency of cancer cells to lose proficiency in one or more repair systems, there is reason to hope that at least for select classes of tumors, such sensitization might be induced selectively in tumor cells, while having less effect on fully repair-competent normal cells.
